# Light Drone-Based Application to Assess Soil Tillage Quality Parameters

**DOI:** 10.3390/s20030728

**Published:** 2020-01-28

**Authors:** Roberto Fanigliulo, Francesca Antonucci, Simone Figorilli, Daniele Pochi, Federico Pallottino, Laura Fornaciari, Renato Grilli, Corrado Costa

**Affiliations:** Consiglio per la Ricerca in Agricoltura e L’analisi Dell’economia Agraria (CREA)-Centro di Ricerca Ingegneria e Trasformazioni Agroalimentari, Via della Pascolare 16, 00015 Monterotondo, Rome, Italy; roberto.fanigliulo@crea.gov.it (R.F.); francesca.antonucci@crea.gov.it (F.A.); simone.figorilli@crea.gov.it (S.F.); daniele.pochi@crea.gov.it (D.P.); laura.fornaciari@crea.gov.it (L.F.); renato.grilli@crea.gov.it (R.G.); corrado.costa@crea.gov.it (C.C.)

**Keywords:** digital agriculture, precision agriculture, UAV, laser profile meter, soil surface roughness, cloddiness

## Abstract

The evaluation of soil tillage quality parameters, such as cloddiness and surface roughness produced by tillage tools, is based on traditional methods ranging, respectively, from manual or mechanical sieving of ground samples to handheld rulers, non-contact devices or Precision Agriculture technics, such as laser profile meters. The aim of the study was to compare traditional methods of soil roughness and cloddiness assessment (laser profile meter and manual sieving), with light drone RGB 3D imaging techniques for the evaluation of different tillage methods (ploughed, harrowed and grassed). Light drone application was able to replicate the results obtained by the traditional methods, introducing advantages in terms of time, repeatability and analysed surface while reducing the human error during the data collection on the one hand and allowing a labour-intensive field monitoring solution for digital farming on the other. Indeed, the profilometer positioning introduces errors and may lead to false reading due to limited data collection. Future work could be done in order to streamline the data processing operation and so to produce a practical application ready to use and stimulate the adoption of new evaluation indices of soil cloddiness, such as Entropy and the Angular Second Moment (ASM), which seem more suitable than the classic ones to achieved data referred to more extended surfaces.

## 1. Introduction

Methods for the assessment of soil tillage quality parameters are often based on traditional practices like manual or mechanical sieving of ground samples for the determination of soil cloddiness produced by tillage tools. Sometimes, for in-soil superficial roughness, the quality of measurement has been increased by introducing instruments and technics of Precision Agriculture, like the laser meters used to scan the soil surface profile.

These methods are slow, hard-working and, anyway, inaccurate, particularly when they refer to large areas, for they depend on the number of samples/measurements carried out.

They can be replaced by multirotor Unmanned Aerial Vehicles (UAV), allowing collection of field data instantly available for evaluations [1], representing a first step towards Digital Agriculture [2]: the collection of aerial data and their processing by means of proper software can be usefully employed in the assessment of tillage quality parameters, with the aim of evaluating the field performance of farm equipment. Such an approach will contribute to more rigorous determination of the operating characteristics of each tractor-machine coupling and of the associated energy requests. This will allow farmers to compare different implements and tillage methods, helping them in the choice of the most useful ones to their agronomic needs, with the aim of obtaining both a good work quality and energy savings [3]. At CREA (Monterotondo, Italy) were carried out the tests to assess the performance of soil tillage machines in accordance with a specific protocol proposed by ENAMA (Italian Agency for Agricultural Mechanization), drawn up by national experts in the sector of the agricultural engineering and based on the current international reference standards (EN, ISO, ASABE). This protocol, characterized by methodological strictness, defines, in detail, the methodology to be followed in performing field tests to assess the main dynamic-energy parameters that characterize the performance of each operating machine [4]. Moreover, the test protocol is recognized in Europe through the ENTAM (European Network for Testing of Agricultural Machinery) which represents a network of as many as 11 European test centers that have signed an agreement for the harmonization of test procedures and the mutual recognition of test reports.

One of the main parameters for the evaluation of agricultural machineries, depending on the environmental conditions, is the qualitative analysis of the work performed (e.g., the action carried out into the soil by the machines working tools). At first, the ENAMA test protocol identifies a series of parameters (soil texture and moisture, dry bulk density, Cone Index) that analyses and classify the substrate on which the machines will be tested and subsequently lists a series of indices that define the quality of the tillage, including the soil surface roughness index [5], the clod size distribution and the clod-breaking index [6].

Soil roughness and cloddiness affect tillage practices and seedbed preparation, surface depression storage, water infiltration, overland flow velocity and runoff processes on cultivated soil. Generally, tillage increases soil roughness and cloddiness when compared to the no-tillage system (grassed soil) while the rain is the main factor that causes its decrease [7]. Thus, soil conservation systems are preferably employed, also to reduce soil erosion [8].

Soil roughness is a measure of the variations in surface elevation and is intended as the aggregate of peaks and depressions present on the soil surface, which may range from few millimeters to several decimeters. The most commonly used methods to measure the surface roughness ranging from handheld rulers to non-contact devices. The firsts consist of a lowered row of pins onto the surface (which measure the elevations) or a given length chain [9] placed across a surface (whose covered horizontal distance will decrease as the roughness increases). These methods are cumbersome, time-consuming and break soil clods. The second group of instruments consist of non-disruptive probes that are not in contact with the soil: laser probes [10,11] or acoustic probes [12]. Profile meter with laser-scanning device measure surface roughness at high resolution, with grid spacing down to millimeter scales, and allow large amounts of data to be acquired. They ensure the high accuracy and reproducibility through the measurement of the time elapsed between the emission of a vertical laser beam towards the surface and the receiving of the reflected beam. However, even such systems are time-consuming and although several data are acquired, these lasts are relative to the single lines analyzed while an imaging 3D technique could give information regarding the whole field plot. Indeed, the results obtained with conventional laser probes could be analyzing a portion of field with specific texture not representative of the whole area under study, unless many measurements are made with a considerable waste of time.

The action of the working tools of soil tillage machines determines the breaking of the soil with the formation of clods, of variable dimensions depending on the type of tillage (primary or secondary). Excessive cloddiness requires further interventions by other operating machines, which increase production costs and degrade the soil structure. The classic method used for separating the soil clods with different dimensions involves the use of cylindrical sieves with a base consisting of a plate with circular holes. Sieving can be manual, by shaking one sieve at a time, or mechanical, by an electromechanical shaker with stacked sieves. In this context, the use of the UAV could increase the reliability of operations, allowing a labor-intensive field monitoring promising solution for digital farming [1].

The aim of this study was to compare traditional methods of soil roughness and cloddiness assessment (laser profile meter and manual sieving), with light drone RGB 3D imaging techniques to evaluate different tillage methods (ploughed, harrowed and grassed).

## 2. Materials and Methods

### 2.1. Experimental Setup for Roughness and Cloddiness Assessment

The roughness of a surface can be expressed through an index which describes the profile of the soil by means of the elevation data. Many researchers have proposed indices to express the surface roughness [13,14], usually based on the standard deviation of sampled height measurements describing the vertical component of the roughness. Grant et al. [15] proposed a roughness index, σ_r_, calculated by means of the following relation:

(1) $$\sigma r={\left\{\frac{1}{N-n-1}{\sum_{i=a+1}^{N-a}\left[\left(yi-\bar{yi}\right)-\left(\bar{yi-\bar{yi}}\right)\right]}^{2}\right\}}^{1/2}$$

where

N is the total number or values collected on a pre-defined distance;
y_i_ is the elevation [mm];
a = number of values before and after y_i_ used for calculating ȳ_i_ (running mean);
n = 2a + 1 is the number of values used for calculating ȳ_i_;
ȳ_i_ is the mean elevation (mm) referred to progressive intervals the length of which is M = 2aDx (mm);
Dx is the horizontal distance between two measurements (10 mm in our case).

The accuracy of the measurements increases with the number of replications and the average values should be accompanied by the coefficient of variation.

**Figure 1:**
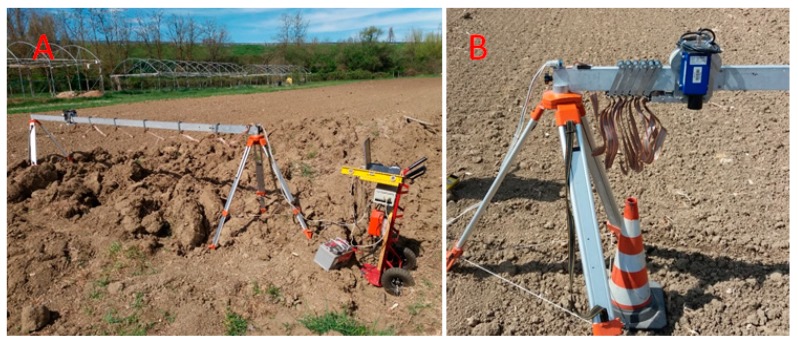
(**A**) Laser microreliefmeter used for determining the surface roughness. On the left the supply and control unit. (**B**) View of the laser sensor moving on a metallic rail.

In the test activity carried out at CREA (Monterotondo, Italy), the surface roughness index is determined, immediately after the soil tillage operation, transversally to the direction of work, by means of an in-house designed and develop profile meter [16]. This instrument consists of a laser sensor (Leica Geosystem Disto, Heerbrugg, Switzerland) which measures its distance from the ground as it moves on a horizontal aluminium rail placed above the tilled strip, by means of a step-by-step electric motor, drawing the surface profile of the ground (Figure 1).

The rail is fixed on two tripods and its length must suffice to cover the entire working width of the machines to be tested. A 4.0 m long rail was used in this study. A personal computer, placed on a manually moved trolley, collected and processed the data (interval between two readings: 10 mm), by means of a software program (in Microsoft Visual Basic 6.0) which controls the movement of the laser probe and the sampling rate per unit of distance. The profile meter was powered by a 12V battery.

The relief of the profiles, in the same point, before and after the execution of an operation (e.g., a secondary tillage), provided, respectively, the roughness indices σ_r1_ and σ_r2_ (based on the calculation of the standard deviation of the detected heights series). These indices enable the calculation of the roughness reduction degree, RRD, resulting from the secondary tillage, as follows (Equation (2)):

(2) $$\mathrm{RRD}\;\left(\%\right)=100\frac{\left(\sigma_{r1}-\sigma_{r2}\right)}{\sigma_{r1}}$$

Other parameters used to describe the quality of the work are the clod size distribution and the clod-breaking index. The cloddiness is based on the determination of the size distribution of the soil aggregates withdrawn from a 0.5 m side square trench dug to the entire working depth, after 20 min drying (at least). Then, the clods are divided into six size classes and weighted. An index (I_ai_), ranging from 0 for the biggest class to 1 for the smallest class, is attributed to each class. The cloddiness results as the percentage of each size class mass referred to total mass of the sample [17]. From the cloddiness, the clod-breaking index (I_a_) is calculated as follows (Equation (3)):

(3) $$I_{a}=\sum_{i=1}^{6}\frac{M_{i}\cdot I_{ai}}{M_{t}}$$

where M_i_·I_ai_ is the product of the index assigned to a clod size class and the mass (kg) of ground belonging to the same class; M_t_ is the total mass of the sample (kg).

### 2.2. Light Drone and Image Acquisition

For the investigation of both soil profile and cloddiness in comparison with the traditional techniques, the light drone UAV DJI™ SPARK™ was employed. Images were taken on 9 April 2019 using the drone lightened to a weight < 300 g according to the Italian Civil Aviation Authority (ENAC) regulation (“Aeromobili a pilotaggio remoto”, Edition 2, Amendment 4, Art. 12, comma 5. May 5th, 2018) on the use of drones without a license. Specifications of this Unmanned Aerial Vehicle (UAV) are described in Table 1. The UAV flew over the experimental field using a planned waypoints mission prepared using the open source software Mission Planner (License GPLv3). The software was used to plan the surface to acquire respecting a series of parameters such as correct images Overlap/Sidelap, ground resolution, image acquisition sync scheduling and flight time. The app used to control the flight and pilot the UAV was Litchi Android App that can load waypoints point from delimited csv file for predefined mission flight.

The digital camera included in the UAV was used to collect still images every 2 S using a shutter speed of 1/2000 s and 100 ISO of sensitivity. Details of the camera technical specifications are described in Table 1. Images were collected using the UAV with the digital camera at 0.5 m s^−1^ at 3 m above ground level (AGL). The details of the experimental flight are shown in Table 2. The images were acquired based on a time-lapse function of the RGB camera vertically oriented that took one image every 2 S ensuring around 75% overlapping ratio. It was used a sidelap of 70%.

**Figure 2:**
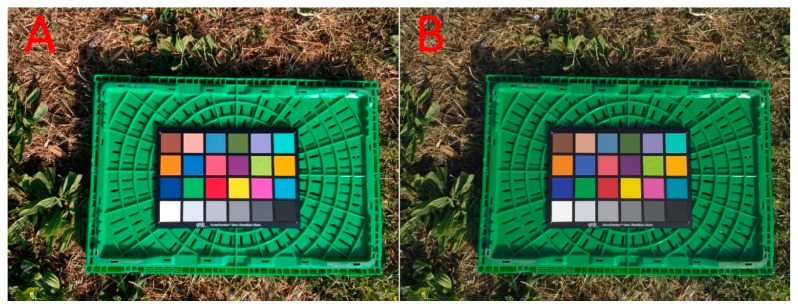
(**A**) Original acquired image from light drone DJI™ SPARK™ with the color checker GretagMacbeth (24 patches) and (**B**) the resulting calibrated one.

At the beginning of the flight on the terrain an image including a color checker GretagMacbeth (24 patches) was acquired. Based on the a priori known color checker patches values all images were calibrated following the Thin-Plate Spline interpolation function [18] in the red, green, blue (RGB) space following the procedure of Menesatti et al. [19] developed in MATLAB (rel 7.1; Mathworks, Natick, MA, USA). This method aims to minimize the effects, for example of the illuminants, camera characteristics and settings. In this procedure, the measured ColorChecker sRGB coordinates within each image were warped (transformed) into the reference coordinates of the same ColorChecker. This transformation was performed through the TPS interpolation function, for the three-dimensional space. Figure 2 shows the original acquired image (A) and the resulting calibrated one (B).

### 2.3. Profile Meter Acquisition and Soil Sieving

The tests were carried out in the farm of CREA (Monterotondo, Rome, Italy), on a flat surface plot (< 1% slope). The soil was classified as silty-clay (clay 543 g kg^−1^, silt 434 g kg^−1^, sand 23 g kg^−1^), according to the USDA soil classification system [20]. The experimental plot had been ploughed earlier with a three-furrow reversible mouldboard plough and subsequently harrowed with a 4 m rotary harrow.

The field setup before measurements process of the surface roughness includes transporting the profile meter to the experimental plot and the following pre-adjusting operations of the apparatus:

− The rail on which the laser sensor runs is placed transversally to the working direction on the tripods whose heights are adjusted so that the rail is perfectly horizontal; when more measurements are needed on the same ground portion subsequent to different operations, the tripods are left on place, the rail is removed during the operations and then restored in its initial position.
− A reference mark is placed on the ground under the starting point of the laser sensor. As described for the tripods, it is left on place undisturbed during subsequent measurements. Thus, the first measured distance represents the reference value, e.g., when assessing roughness variations in the same ground portion before tillage and after primary and secondary tillage.

Five replicates of soil profiles, randomly chosen in the test plots (ploughed, harrowed and grassed), were parallelly collected, 1 m from each other. The soil profile of each replicate was described by the 400 z values measured by the laser sensor.

The cloddiness of the ploughed and harrowed plots were acquired until the implements working depth (0.40 m and 0.20 m respectively). Five hand-operated standard sieves (Controls, Milan, Italy) with different diameter of the circular holes (200, 100, 50, 25 and 10 mm) were used to identify six soil size classes: <200 mm, 200 ÷ 100 mm, 100 ÷ 50 mm, 50 ÷ 25 mm, 25 ÷ 10 mm, < 10 mm, respectively. The soil mass included in each class was weighed using a field balance, with a maximum capacity of 100 kg (50 g resolution). A set of three replicates of soil cloddiness was collected, randomly chosen in the test plots (ploughed, harrowed).

### 2.4. 3D and Ortho Image Reconstruction

**Figure 3:**
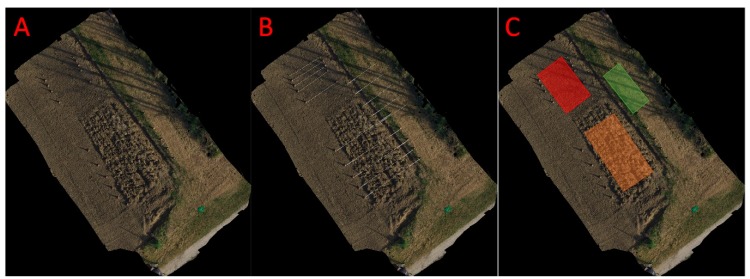
The reconstructed orthoimage (**A**) overlapped with the transects (**B**); white dashed lines) and area for the texture parameters extraction (**C**); Orange = ploughed, red = harrowed and green = grassed).

After the acquisition, the 257 pictures collected were analysed to reconstruct the orthoimages with the software “3DF Zephyr” (Zephir 3DFLow 2018, Verona, Italy) [21] through the following steps: project creation selecting the pictures needed; camera orientation and sparse point cloud generation present at high accuracy with the images at 100% resolution (no resize); dense point cloud generation; mesh extraction; textured mesh generation; export outcome files including the Digital Surface Model (DSM), the Digital Terrain Model (DTM) and the orthoimage (Figure 3A).

### 2.5. Transect and Texture Acquisition from Orthoimage and DSM

On the DSM, five transects for each plot (ploughed, harrowed and grassed) corresponding approximately to the profilometer acquisition positioning were manually acquired (Figure 3B). The transect z values were acquired extracting 1000 equi-spaced points. Data from both profilometer and transects were standardized using the following formula:

(4) $$z_{standard}=\frac{z_{i}-mean}{SD}$$

On the DSM, one area for each plot (ploughed, harrowed and grassed) was manually extracted (Figure 3C) and elaborated to obtain texture values. Texture is a measure of the variation of the intensity of a surface, quantifying properties such as smoothness, coarseness and regularity [22]. The hypothesis is to obtain, from the three DSM areas, standard information on the homogeneity of the surface. Two main texture indicators were extracted: Entropy and Angular Second Moment (ASM). To extract this information image was converted from RGB to grayscale using the average method.

Entropy measures the disorder of an image and it achieves its largest value when all elements in P matrix are equal [23]. When the image is not texturally uniform many Grey Level Co-Occurrence Matrix (GLCM) elements have very small values which imply that entropy is very large. Therefore, entropy is inversely proportional to GLCM energy:

(5) $$ENTROPY=-\underset{i}{\sum }\underset{j}{\sum }p\left(i,j\right)\log p\left(i,j\right)$$

Energy, also called Angular Second Moment and Uniformity is a measure of textural Uniformity of an image [24]. Energy reaches its highest value when grey level distribution has either a constant or a periodic form. A homogenous image contains very few dominant grey tone transitions and therefore, the matrix for this image will have fewer entries of larger magnitude resulting in large value for energy feature. In contrast, if the matrix contains many small entries, the energy feature will have a smaller value:

(6) $$ASM=-\underset{i}{\sum }\underset{j}{\sum }{\left\{p\left(i,j\right)\right\}}^{2}$$

Both transects, and area textural values were extracted using ImageJ: plot profile tool for transects and GLCM texture plugin for area textural values.

## 3. Results

**Figure 4:**
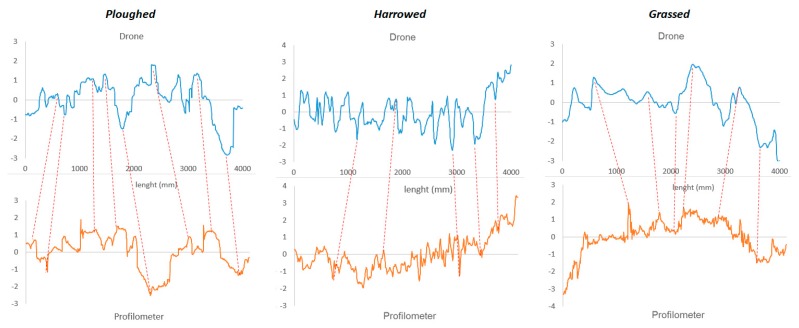
Profiles of the 3 types of soil (ploughed, harrowed and grassed) extracted with both light drone (blue line) and profilometer (orange line). *y*-axes are adimensional.

Figure 4 shows the profiles of the three types of soil (ploughed, harrowed and grassed) extracted with the two methods compared (light drone and profilometer). It is possible to observe a general relationship between the two curves evidenced by red dashed lines. Even if detection methods and sensors are different, a similar trend could be observed. Generally, the drone survey is not exactly possible with the profile of the profilometer (positioning error of few centimeters) since estimated a posteriori relying on approximated ground points. y-axes are adimensional.

**Figure 5:**
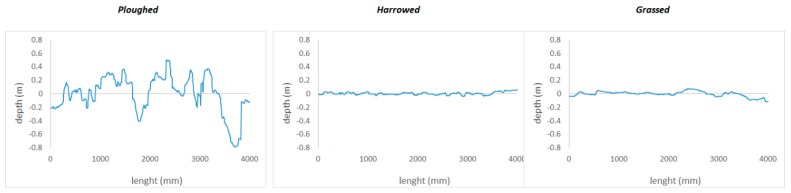
Profiles values extracted with the light drone converted to metric scale of the 3 types of soil (ploughed, harrowed and grassed).

Figure 5 reports the values of the profiles converted to metric scale for the three types of soil (ploughed, harrowed and grassed) extracted with the light drone. The fluctuations are evident for the ploughed soil. For the harrowed, it is possible to observe as the fluctuations are lower and at high frequency, while for the grassed one the trend is much softer than the other two and at lower frequencies.

Table 3 shows the standard deviations for the three different types soil (ploughed, harrowed and grassed) for both profilometer and light drone. These values provide an indication about soil cloddiness. The values were different between the two techniques, however, with a similar trend (greater for ploughed, minor for grassed). The much higher magnitude for the drone values could be due to the kink of sensor used (imaging vs. laser).

In terms of surface analysis, obtained only by light drone imaging, Table 4 shows the values of entropy and ASM for the three soil types and of the I_a_ (clod breaking index) resulting from the ploughing and harrowing. High entropy and low values of ASM are given for ploughed soil which indicates increased irregularity due to the mainly variable quotas as confirmed by the values in Table 3. The values of I_a_ and ASM resulting from the two tillage operations have similar trend. Ploughed soil showed higher entropy and lower ASM with respect to harrowed and grassed ones.

## 4. Discussion

Factors like clods size and distribution on the soil surface are influenced by agricultural operations and they determine crop yield [25]. Detailed and accurate information on soil surface roughness and cloddiness are a relevant parameter to evaluate the performance of soil tilling machines, tillage methods and seedbed preparation [17]. There are relatively accurate and robust techniques able to estimate the effect of soil random roughness on machines performances. As reported by Bögel et al. [26] many measurements devices were adopted to quantify soil surface from mechanical solutions (e.g., pin meter and chain ruler), to laser scanner, cameras or acoustic instrumentations. The method commonly used for determining the degree of cloddiness resulting from tillage operations is the sieve analysis (i.e., the clods diameter is calculated splitting the sample into a given number of fractions) while as an alternative, image analysis techniques or others can be used for the determination of surface roughness [17]. Nowadays, the measurements of surface elevation is done with automated non-contact profile meter, utilize laser technology. On the other hand, limited data are available with respect to the capability of an UAV on estimation of soil surface roughness and cloddiness.

Generally, as reported by Kaźmierowski et al. [27], the soil surface roughness and cloddiness quantification on a large scale (field scale level) is more problematic and challenging than at laboratory scale. Indeed, bigger scales calculations can influence the results. In the present study, the traditional methods (laser profile meter and manual sieving) most used for these purposes have been compared with the recent technology of a light drone after different tillage methods (ploughed, harrowed and grassed).

The technique used demonstrated to be adequate and suitable for the proposed scope producing advantages in terms of both time and, as the results of the study demonstrate, precision that could increase, due to the higher surface that could be analyzed, in relation to the reduction of the human error during the data collection. Indeed, the profilometer positioning introduces errors of few centimeters. In addition, in comparison with static techniques, images taken from the UAV can maintain near-nadir looking geometry over scales of several agricultural fields [26]. The other suitable tools for determining soil roughness and cloddiness (e.g., the terrestrial laser scanning), as pointed out by Milenković et al. [28,29] are not practical and suitable for the analysis of large areas. This is physically not necessarily true but obviously, a not practicable option when come to real life. Indeed, to scan a whole field using punctual techniques will requires a tremendous amount of time while flying over it we can gather information spatialized all over it. Moreover, the lines sectioned from the 3D reconstructions can be position in whichever direction wanted to increase the data heterogeneity.

## 5. Conclusions

A variety of techniques today can be used in order to evaluate the soil tillage quality parameters which are crucial to choose the most suitable tillage methods and machineries on the base of farm specific agronomic needs and size. Indeed, such choices are important to optimize plant growth and resource use. Therefore, an accurate analysis regarding a whole plot could produce advantages for the farmer. Generally, soil roughness and cloddiness affect tillage practices and seedbed preparation, water infiltration, overland flow velocity and runoff processes on cultivated soil. Morever, their assessment is used to evaluate the performance of soil tillage machines. Traditional methods are based on punctual readings and are normally very time consuming in order to achieve reliable assessments of the mentioned parameters if considering the heterogeneity present within a whole field plot.

The study tested a light UAV application in comparison with traditional methods, in order to rapidly obtain a large set of data and to extend the surface analysed potentially to the whole field. The proposed method seems capable to present a general relationship with the traditional methods and to yield results with good accuracy and reproducibility in relation to the value of the surface roughness index (σ_r_), obtained with the profilometer, and of the clod-breaking index (I_a_), gained by the manual sieving. In addition, the introduction of image analysis techniques will probably imply the adoption of new evaluation indices, such as the Entropy or the ASM proposed in this study, which seem more suitable than the classic ones to synthesize the achieved information referred to extended surfaces. This process will require specific studies aimed at defining the correspondence between new and old indices able to compare not only other tillage methods, typical of conservative agriculture, but also other implements, each capable of providing different effects on quality parameters. Moreover, in terms of budget, the developed applications require a reduced budget and, therefore, could be called low-cost. Indeed, the instruments needed (light drone) can be acquired today for few hundred euros. Future work could lead to the streamline of the processing operation in order to produce a practical application ready to use. Nevertheless, the process can be streamlined and automated for easy operation and data analysis. This work represents one step among several nowadays that are shifting the agricultural management from precision farming towards digital farming.

## Figures and Tables

**Table 1:** Specifications of the Unmanned Aerial Vehicle (UAV) DJI™ SPARK™.

Details	Items	Specifications
**Light drone**	Weight	297 g
	Dimensions	143 ×143 × 55 mm
	Max speed	50 km/h
	Satellite positioning systems	GPS/GLONASS
**Digital camera**	Camera Focal Lenght	4.5 mm
	Sensor dimensions (WxH)	6.17 × 4.56 mm
	Sensor Resolution	12 megapixels
	Image Sensor Type	CMOS
	Capture Formats	MP4 (MPEG-4 AVC/H.264)
	Still Image Formats	JPEG
	Video Recorder Resolutions	1920 × 1080 (1080p)
	Frame Rate	30 frames per second
	Still Image Resolutions	3968 × 2976
**GIMBAL**	Control range Inclination	from −85° to 0°
	Stabilization	Mechanical 2 axes (inclination, roll)
	Obstacle detection distance	0.2–5 m
	Operating environment	Surfaces with diffuse reflectivity (> 20%) and dimensions greater than 20 × 20 cm (walls, trees, people, etc.)
**Remote Control**	Operating Frequency	5.8 GHz
	Max Operating Distance	1.6 Km
**Battery**	Supported Battery Configurations	3S
	Rechargeable Battery	Rechargeable
	Technology	lithium polymer
	Voltage Provided	11.4 V
	Capacity	1480 mAh
	Run Time (Up To)	16 min
	Recharge Time	52 min

**Table 2:** Experimental unmanned aerial vehicle (UAV) flight details.

Flight date	Image Number	Flight Altitude (m)	Flight Speed (m s^−1^)	Ground Resolution (cm)	Illumination
Apr. 9, 2019	257	3	0.5	0.01	Natural light

**Table 3:** Standard deviation values (in mm) for profilometer and light drone plots for the 3 types of soil (ploughed, harrowed and grassed) and roughness reduction degree (RRD; ploughed/harrowed).

Type of Soil	Profilometer	Drone
Grassed	0.18	0.04
Ploughed	0.54	0.27
Harrowed	0.11	0.02
RRD	79.62	92.59

**Table 4:** Values of I_a_ (laser profilometer), entropy and ASM light drone areas for the 3 types of soil (plowed, harrowed and grassed).

Type of Soil	I_a_	Entropy	ASM
Grassed	/	4.478	0.017
Ploughed	0.45	5.391	0.007
Harrowed	0.84	4.852	0.011
